# A Rare Case of Dissecting Superior Mesenteric Artery Aneurysm in Granulomatosis with Polyangiitis

**DOI:** 10.3400/avd.avd.cr.23-00050

**Published:** 2023-10-13

**Authors:** Shinichi Tanaka, Takahiro Ohmine, Takashi Maeda

**Affiliations:** 1 Department of Surgery, Hiroshima Red Cross Hospital and Atomic-bomb Survivors Hospital, Hiroshima, Hiroshima, Japan

**Keywords:** superior mesenteric artery aneurysm, granulomatosis with polyangiitis, stent-graft

## Abstract

An asymptomatic dissecting superior mesenteric artery (SMA) aneurysm in granulomatosis with polyangiitis (GPA), historically termed Wegener’s granulomatosis, is rare. We herein describe a 68-year-old man who was diagnosed with GPA based on a high level of proteinase 3 (PR3)-antineutrophil cytoplasmic antibody (ANCA). One year after remission of GPA, the patient developed pyelonephritis, and his PR3-ANCA level increased again. Computed tomography showed a rapid increase in the size of the dissecting SMA aneurysm. The patient underwent successful endovascular stent-graft repair. At the time of this writing, 3 years had passed since the surgery and the clinical course was good.

## Introduction

Abdominal visceral aneurysms are relatively rare. Among this type of aneurysm, superior mesenteric artery (SMA) aneurysms are particularly rare.^1)^ Granulomatosis with polyangiitis (GPA) is a small-vessel systemic antineutrophil cytoplasmic antibody (ANCA)-associated vasculitis primarily involving the respiratory tract and kidneys.^2)^ Although there have been reports of GPA being complicated by aneurysms in small- and medium-sized vessels, no reports have described GPA complicated by a dissecting SMA aneurysm. We herein report a case in which we successfully performed endovascular stent-graft placement to treat an asymptomatic dissecting SMA aneurysm in a patient with GPA.

## Case Report

**Figure 1:**
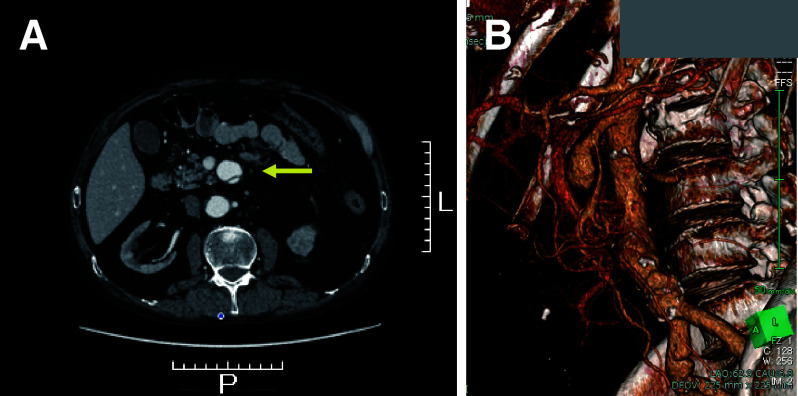
Fig. 1 (**A**) Preoperative enhanced CT showed a 2.4-cm dissected SMA aneurysm (arrow). (**B**) Three-dimensional CT. CT: computed tomography; SMA: superior mesenteric artery.

A 68-year-old man was diagnosed with GPA at another hospital based on a history of refractory cough, an elevated inflammatory response and positive proteinase 3 (PR3)-ANCA on blood tests, and granular shadows in the lung fields. He achieved remission after treatment with steroid pulse therapy, high-dose steroids, and oral cyclophosphamide hydrate tablets, and he thereafter received azathioprine as maintenance therapy. Two years after the diagnosis of GPA, his renal function worsened and he began hemodialysis. Six months after the introduction of hemodialysis, the patient developed pyelonephritis and his PR3-ANCA level increased from 5.1 U/mL to 88.6 U/mL (reference range: <3.5 U/mL). Computed tomography (CT) revealed an SMA aneurysm that had rapidly enlarged from 11 to 24 mm in 1 year (**Fig. 1**). The patient had no history of blunt or sharp trauma, such as that induced by a traffic accident, catheterization, or treatment of abdominal arteries during this period.

The patient underwent endovascular repair using a stent-graft after consideration of his comorbidities and the anatomical characteristics of the aneurysm. The brachial artery approach was chosen to deploy the stent-graft. A 4-Fr sheath was introduced through right femoral access using a pigtail catheter. A 9-Fr sheath was introduced through the left brachial artery after cutting down. A 6-Fr, 45-cm Destination EX sheath (Terumo, Tokyo, Japan) was introduced into the 9-Fr sheath. A 4-Fr multipurpose catheter (Merit Medical, South Jordan, UT, USA) with a 0.035-inch Radifocus guidewire (Terumo) was introduced to the SMA.

**Figure 2:**
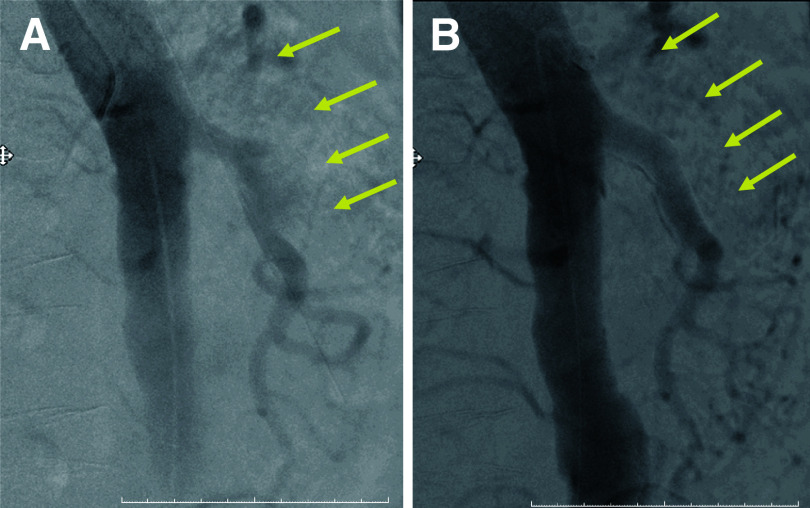
Fig. 2 (**A**) Pre-implantation of stent-graft angiography showed SMA aneurysm (arrows). (**B**) Completion angiography showed exclusion of the aneurysm preservation of the middle colic artery as well as the distal branches of the SMA with no endoleak (arrows). SMA: superior mesenteric artery.

The guidewire was exchanged for a 0.035-inch, 260-cm stiff guidewire. The sheath via the brachial artery was exchanged for a 12-Fr sheath, and the sheath was engaged in the SMA. The stent-graft diameter was 1 mm greater than that of SMA, and the stent-graft length was at least 10 mm at the distal and proximal margins on preoperative CT. A 10- × 50-mm stent-graft (Viabahn; W.L. Gore & Associates, Flagstaff, AZ, USA) was deployed from the orifice of the SMA to the orifice of the first ileal branch. We performed angiography from the pigtail catheter of the femoral access to avoid jailing the ileal branch. The post-stenting angiogram of the SMA demonstrated preservation of the distal branches of the SMA with no endoleak (**Figs. 2A** and **2B**). The postoperative course was uneventful.

**Figure 3:**
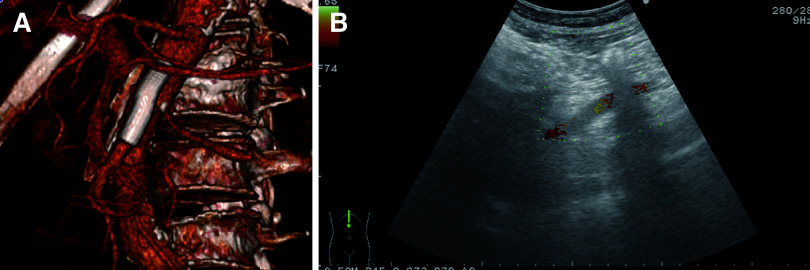
Fig. 3 (**A**) Postoperative CT showed exclusion of the aneurysm, patent stent-graft, and preservation of the middle colic artery as well as the distal branches of the SMA with no endoleak. (**B**) Postoperative echo at 3-year follow-up showed patent stent-graft of SMA. CT: computed tomography; SMA: superior mesenteric artery.

At the time of this writing, the patient had remained free of symptoms for 3 years after the procedure, and follow-up CT (**Fig. 3A**) and ultrasound (**Fig. 3B**) confirmed successful exclusion of the aneurysm with no endoleak. The patient was prescribed oral clopidogrel (75 mg/day) and aspirin (100 mg/day) for 6 months. Thereafter, only aspirin was prescribed for the remainder of the patient’s life.

## Informed consent

The patient provided his written informed consent for the publication of the details of his case.

## Discussion

Among abdominal visceral aneurysms, SMA aneurysms are particularly rare.^1)^ The causes of aneurysms include arteriosclerosis, congenital vascular anomalies, vasculitis, pancreatitis, and traumatic injury; some aneurysms are iatrogenic.^3)^ GPA is a systemic necrotizing sarcoid vasculitis, and its clinical and pathological features include necrotizing sarcoidosis of the upper and lower airways and necrotizing crescentic nephritis of the kidneys.^2)^ The PR3-ANCA level in patients with GPA reflects the disease activity and is considered a treatment indicator.^4)^ Vasculitis in GPA mainly affects small vessels, and aneurysmal complications are very unusual.^2)^ An asymptomatic dissecting SMA aneurysm in GPA is very rare. Based on the available data in PubMed, we believe that this is the first report of endovascular stent-graft repair of an asymptomatic dissecting SMA aneurysm in a patient with GPA.

The SMA diameter in our patient rapidly increased from 11 to 24 mm within 1 year, and this enlargement was at the same time of elevation of the PR3-ANCA level. Because endovascular treatment was performed in this case, it is difficult to discuss about the pathology of the mechanism of dissecting and aneurysm formation. Although it was suspected that a dissecting aneurysm occurred as a result of exacerbation of vasculitis activity, it is impossible to state a direct causal relationship clearly. There are reports of aneurysms that have formed after the normal PR3-ANCA level in this rare complication of the disease, and such aneurysms may occur independently of the activity of GPA itself.^5)^ Aneurysm formation of any size is possible at any time during the course of GPA, although its incidence is low.^2)^

In accordance with the Society for Vascular Surgery clinical practice guidelines,^6)^ we performed endovascular repair instead of laparotomy because of the patient’s anatomical characteristics. Jiang et al.^7)^ stated that the three indications for endovascular treatment were as follows: the stent-graft should not obstruct the branch entering the small intestine, the aneurysm should not be too large and should allow for an adequate landing zone, and the aneurysm should not be infectious. Our case met all of these conditions. Patients who do not meet the above conditions should undergo surgical revascularization.^8)^

An innovation of treatment by stent-grafts is to secure two access routes: one with a pig-tail catheter for angiography in real time via the femoral artery and the other for deployment of the stent-graft via the brachial artery.^7)^ Angiography before deployment via the femoral access catheter can facilitate more accurate localization of the stent-graft and avoid covering extra branch arteries.^7)^

## Conclusion

We have herein reported a rare case of a dissecting SMA aneurysm in a patient with GPA treated with endovascular stent-graft repair.

## Acknowledgments

We thank Angela Morben, DVM, ELS, from Edanz (https://jp.edanz.com/ac), for editing a draft of this manuscript.

## Disclosure Statement

All authors have no conflicts of interest.

## Author Contributions

Study conception: ST

Data collection: ST

Analysis: ST

Investigation: ST

Manuscript preparation: ST

Funding acquisition: none

Critical review and revision: all authors

Final approval of the article: all authors

Accountability for all aspects of the work: all authors.
